# The prognostic value of modified Glasgow Prognostic Score in pancreatic cancer: a meta-analysis

**DOI:** 10.1186/s12935-020-01558-4

**Published:** 2020-09-22

**Authors:** Huan Zhang, Dianyun Ren, Xin Jin, Heshui Wu

**Affiliations:** 1 Department of Pancreatic Surgery, Union Hospital, Tongji Medical College, Huazhong University of Science and Technology, No.1277 Jiefang Avenue, Wuhan, 430022 Hubei China; 2 Sino-German Laboratory of Personalized Medicine for Pancreatic Cancer, Union Hospital, Tongji Medical College, Huazhong University of Science and Technology, Wuhan, 430022 China; 3 Cancer Center, Union Hospital, Tongji Medical College, Huazhong University of Science and Technology, Wuhan, 430022 China

**Keywords:** Pancreatic cancer, Modified Glasgow Prognostic Score (mGPS), Prognostic value, Meta-analysis

## Abstract

**Background:**

Several studies were conducted to explore the prognostic value of modified Glasgow Prognostic Score (mGPS) in pancreatic cancer, which reported contradictory results. The purpose of this meta-analysis was to summarize and further investigate the correlation between mGPS and overall survival (OS) in pancreatic cancer.

**Methods:**

A systematic literature search was performed in PubMed, EMBASE, ISI Web of Science, Cochrane library databases and OVID to identify eligible studies published from Jan 1, 2011 to June 20, 2020. Pooled hazard ratios (HRs) with corresponding 95% confidence intervals (CIs) were used to detect the prognostic significance of mGPS in patients with pancreatic cancer.

**Results:**

A total of 222 non-repetitive studies were identified, and 20 related studies that explored the association between survival outcomes and mGPS in pancreatic cancer patients were finally enrolled in this meta-analysis. The results showed a significant correlation between high level of mGPS and poor OS (HR = 1.50, 95% CI 1.20–1.89, P < 0.0001). Similar results were observed in the subgroup analyses based on the treatment regimen and research region.

**Conclusions:**

Our study suggested the close association between poor prognosis and high level of mGPS, which will be helpful for future clinical applications in patients with pancreatic cancer.

## Introduction

Pancreatic cancer, one of the most devastating human malignancies, is the fourth leading cause of cancer death, and the 5-year survival rate for all stages of pancreatic cancer is as low as 6–8% [1]. It was estimated that approximately 47,050 patients would die of this disease in the United States in 2020 [2]. Due to the trend of early metastasis, the 5-year survival rate of pancreatic cancer was only 8% [2]. In addition, pancreatic cancer is projected to surpass breast, prostate, and colorectal cancers to become the second leading causes of cancer-related death by 2030 [3].

As a major component of the tumor microenvironment, the role of cells and inflammation mediators in tumor invasion and metastasis was widely recognized mediating proliferation and survival of malignant cells, stimulating angiogenesis and metastasis, subverting adaptive immunity, and reducing response to hormones and chemotherapy [4, 5]. Recently, several studies have proved the prognostic significance of multiple prognosis-related scoring rubrics in a variate of cancers based on systemic inflammation, such as neutrophil-to-lymphocyte ratio (NLR), platelet-to-lymphocyte ratio (PLR), C-reactive protein (CRP) and CRP-to-albumin ratio (CAR), as well as modified Glasgow Prognostic Score (mGPS) [6–9]. Among these, mGPS, consisting of the level of serum CRP and albumin, was considered to have similar prognostic ability to performance status [10]. The mGPS ranges from 0 to 2: patients with both CRP increase (> 10 mg/L) and hypoalbuminemia (< 35 g/L) is defined as a score of 2; patients with normal CRP level and albumin level is defined as a score of 0; patients with only increased CRP level is defined as a score of 1 [10].

The prognostic value of mGPS has been confirmed in a variety of solid tumors, such as small cell lung cancer, colorectal cancer, gastric cancer, and ovarian cancer [11–14]. Several studies proved that mGPS was one of the most important determinants of overall survival (OS) in pancreatic cancer patients [15, 16], but others showed contradictory results [17, 18]. Therefore, the exactly prognostic value of mGPS remained to be further confirmed. Our study aimed to investigate the prognostic significance of mGPS in patients with pancreatic cancer.

## Methods

### Literature search strategy

Two authors (Zhang and Ren) independently used the following databases: PubMed, EMBASE, ISI Web of Science and Cochrane library databases to obtain relevant articles (published from Jan 1, 2011 to June 20, 2020). We used the following combined text and Medical Subject Headings (MeSH) as follows: terms: “Pancreatic Neoplasms”. The complete literature search used for PubMed was: ((((((((((Pancreatic Neoplasm[Title/Abstract]) OR Neoplasm, Pancreas[Title/Abstract]) OR Pancreas Neoplasm[Title/Abstract]) OR Pancreatic Cancer[Title/Abstract]) OR Pancreas Cancer[Title/Abstract]) OR Cancer of the Pancreas[Title/Abstract]) OR pancreatic ductal adenocarcinoma[Title/Abstract]) OR PDAC[Title/Abstract]) OR “Pancreatic Neoplasms“[Mesh])) AND ((modified Glasgow Prognostic Score[Title/Abstract]) OR mGPS[Title/Abstract]). Furthermore, the references in these eligible articles were also manually reviewed to identify potentially relevant studies.

### Inclusion and exclusion criteria

Eligible studies must meet the following criteria: (1) the diagnosis of pancreatic cancer was confirmed by pathological methods; (2) the relationships between mGPS and OS or other survival parameters were investigated for patient with pancreatic cancer; (3) the mGPS were calculated using a recognized standard method; (4) hazard ratio (HR) and 95% confidence interval (95% CI) of OS or other survival parameters were reported or could be calculated by Tierney’s method [19] ; (5) studies were published as full-text articles in English; (6) studies were considered qualified if they met all of the following requirement: unrelated articles, conference abstracts, letters, reviews, case reports and studies without enough data were excluded; (7) if multiple studies were performed in the same center and the samples were overlapped, the study with the largest sample size was included. Whereas, the exclusion criteria were as follows: (1) duplicated articles; (2) experimental studies; (3) case reports, editorial, letters, review articles, and meta-analyses, conference abstracts; (4) studies with unavailable data and irrelevant articles (5) studies with insufficient prognostic outcomes.

### Data extraction and quality assessment

All data were extracted from eligible studies by two independent investigators (Zhang and Ren). Any disagreement between the two investigators was settled by discussion. The following information from each study was extracted: first author, country of the population enrolled, year of publication, sample size, patient characteristics (age, gender, tumor stage), outcome parameter, therapy strategy, mean follow-up, research duration and so on. The quality of included studies was assessed by Newcastle–Ottawa Scale (NOS), including the following aspects: representativeness of the exposed cohort, selection of the non-exposed cohort, ascertainment of exposure and demonstration that outcome of interest was not present at start of study; comparability of cohorts on the basis of the design or analysis; assessment of outcome, follow-up time was sufficient enough for results to occur and adequacy of follow-up of cohorts [20].

### Statistical analysis

Pooled HRs with corresponding 95% CIs were used to evaluate the association between the mGPS and OS. Heterogeneity among studies was assessed using chi-square-based Higgins I^2^ statistic [21], and I^2^ > 50% indicated significant heterogeneity. The fixed effect model was used only when I^2^ < 50%, otherwise a random effect model was executed. HRs and 95% CIs were utilized as the effect value to assess the association between mGPS and OS in pancreatic cancer. The Begg’s funnel plot was used to assess the presence of potential publication bias by plotting the effect sizes calculated from individual studies examining the association between HR and standard error (SE) of OS. Publication bias was assessed by the Begg’s test [22], with P > 0.05 implying no significant publication bias. All statistical analyses were performed by STATA version 15.0 (StataCorp, College Station, TX, USA).

## Results

### Study selection and characteristics

**Fig. 1:**
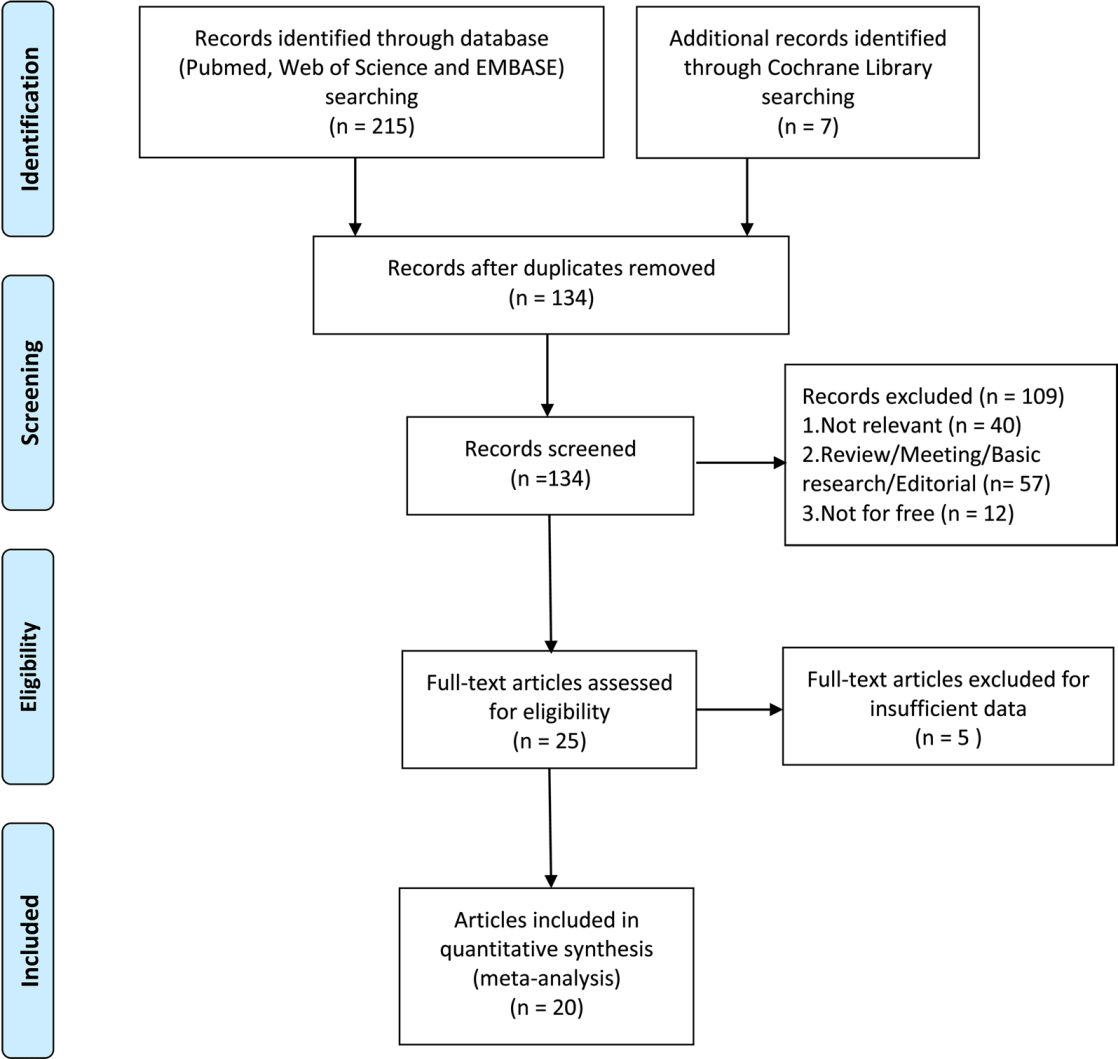
Flow diagram of selecting relevant published works

A total of 222 papers were initially retrieved from PubMed, Web of Science, EMBASE and Cochrane library database. As shown in Fig. 1, the literature search process was summarized in the flow diagram according to Preferred Reporting Items for Systematic Reviews and Meta-analyses (PRISMA) [23]. Ultimately, 20 studies [8, 15–18, 24–38] including 6512 patients were enrolled in this meta-analysis after excluding ineligible studies.

Basic characteristics and main outcomes of the included 20 studies were shown in Table 1. These studies were published from 2011 to 2020, with a research duration ranging from 2000 to 2017. The sample size of these studies ranged from 47 to 1347, with a total number was 6512. As for the therapeutic methods, patients in 14 studies received chemotherapy, and pancreatectomy was chosen in another 6 studies. The NOS score of all included studies were equal or greater than 5, which meant relatively high quality. 16 studies were conducted in Asia (including China, Japan, and South Korea), and 4 studies were performed in western countries (including the United Kingdom, Italy and Australia). Most studies were assessed with multivariate analysis except that 4 studies were only assessed with univariate analysis.

**Table 1:** Basic characteristics of included studies

Study	Year	Country	Sample size/N	mGPS group	Age (year)(%)	Survival analysis	NOS score	Tumor stage	Therapy	Mean Follow-up (month)	Research duration
Partridge et al. [30]	2012	UK	102 (16/20/66)	0/1/2	≤ 72, (50%)	M	6	Advanced	Chemotherapy	–	2006.01–2006.10
Jamieson et al. [32]	2011	UK	135 (74/61)	Low/high	≤ 65, (54%)	M	6	T2/T3	Pancreatectomy	–	2002–2009
Torre et al. [29]	2012	Italy	82 (37/45)	Low/high	–	M	5	I/IV	Chemotherapy	19	2003–2009
Wang et al. [26]	2012	China	177 (115/62)	Low/high	≤ 65, (70.1%)	U, M	6	II/IV	Chemotherapy	31.33	2006–2010
Inoue et al. [25]	2015	Japan	440 (367/49/24)	0/1/2	≤ 65, (40.7%)	U	7	I/IV	Chemotherapy	18.7	2008–2012
Martin et al. [27]	2014	Australia	124 (46/78)	Low/high	–	U, M	7	Locally advanced or metastatic	Chemotherapy	12	2008–2012
Mitsunaga et al. [18]	2016	Japan	141 (79/39/23)	0/1/2	≤ 67, (50%)	M	5	Advanced	Chemotherapy	–	2008–2013
Imaoka et al. [38]	2016	Japan	807 (620/153/34)	0/1/2	< 75, (81.9%)	U	7	Locally advanced or metastatic	Chemotherapy		2001–2013
Wu et al. [8]	2016	China	233 (119/114)	Low/high	< 62, (47.6%)	U, M	5	Advanced	Chemotherapy	–	2011–2014
Kawai et al. [17]	2016	Japan	1347 (1121/115/111)	0/1/2	–	M	6	I/IV	Pancreatectomy	25.3	2001–2012
Yamada et al. [33]	2016	Japan	305 (243/62)	Low/high	≤ 65, (50.1%)	U, M	5	I/IV	Chemotherapy	–	2002–2014
Liu et al. [15]	2017	China	386 (131/242/13)	0/1/2	≤ 65, (35.8%)	U	5	I/IV	Chemotherapy	8.7	2010–2015
Iino et al. [24]	2017	Japan	47 (35/12)	Low/high	–	U, M	7	Locally advanced or metastatic	Chemotherapy	–	2010–2015
Xiao et al. [34]	2017	China	66 (39/27)	Low/high	–	U, M	6	Advanced	Chemotherapy	–	2012–2013
Fujjiwara et al. [35]	2018	Japan	188 (140/21/27)	0/1/2	< 70, (54.3%)	U, M	8	I/IV	Pancreatectomy	–	2000–2015
Ikuta et al. [36]	2019	Japan	136 (131/5)	Low/high	≤ 68, (54.4%)	U	5	I/IV	Pancreatectomy	16.8	2005–2017
Abe et al. [16]	2018	Japan	329 (282/47)	Low/high	< 65, (43.76%)	U, M	6	I/IV	Pancreatectomy	–	1996–2014
Shin et al. [31]	2018	South Korea	1092 (587/353/152)	0/1/2	–	M	6	I/IV	Chemotherapy	21.6	2000–2016
Hwang et al. [28]	2018	South Korea	203 (137/66)	Low/high	≤ 65, (62.1%)	U, M	8	Metastatic or recurrent	Chemotherapy	21.5	2016.01-2016.12
Nakagawa et al. [37]	2019	Japan	172 (157/15)	Low/high	< 70, (49.4%)	U, M	6	I/IV	Pancreatectomy	–	2006–2015

“–” means not available; N means number; U means univariable regression analysis; M means multivariable regression analysis

### Prognostic value of mGPS in pancreatic cancer

**Fig. 2:**
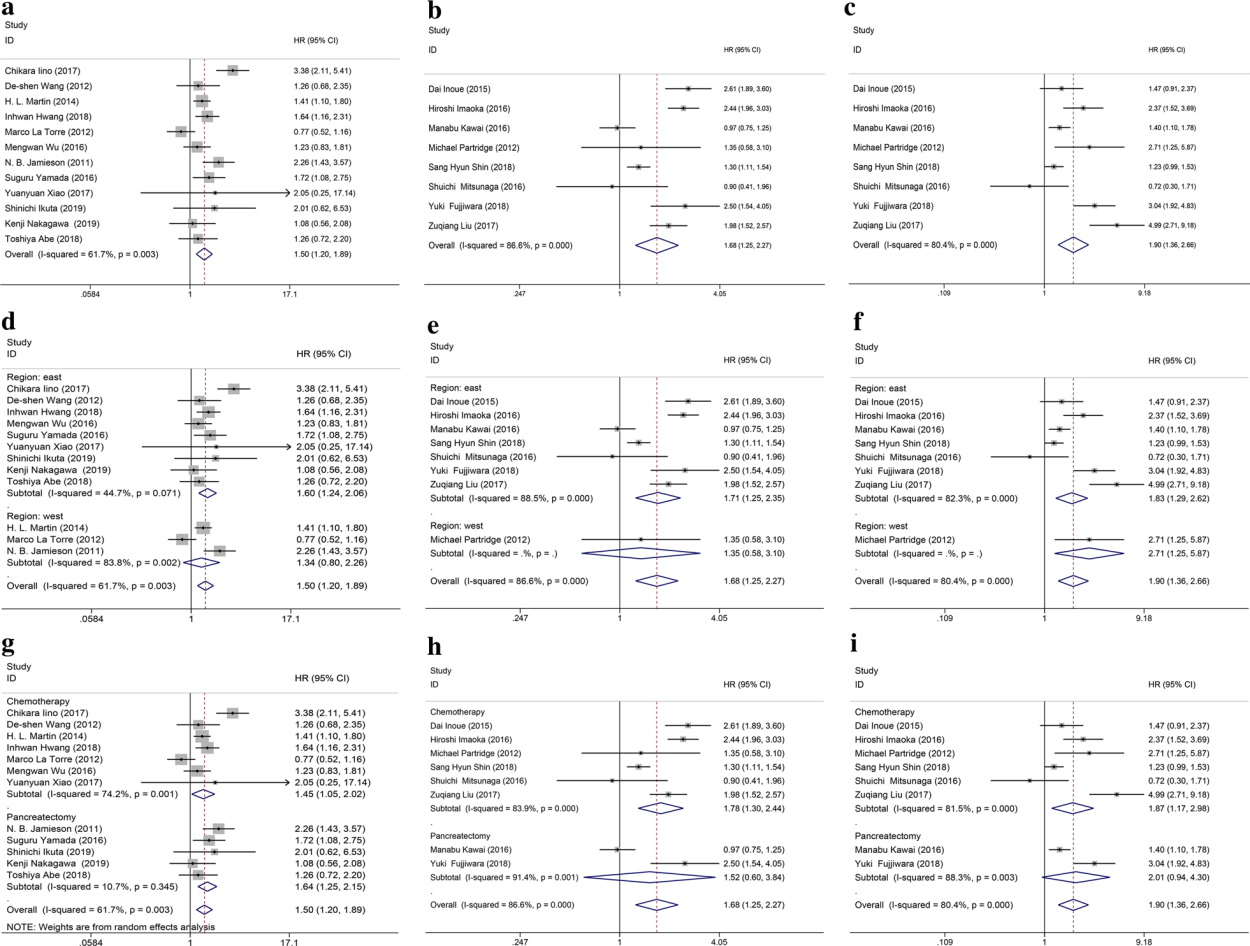
Forest plots regarding the prognostic significance of mGPS in OS. Overall: **a** mGPS high vs. low; **b** mGPS 1 vs. 0; **c** mGPS 2 vs. 0; Subgroup analysis by research region: **d** mGPS high vs. low; **e** mGPS 1 vs. 0; **f** mGPS 2 vs. 0; Subgroup analysis by treatment regimen: **g** mGPS high vs. low; **h** mGPS 1 vs. 0; **i** mGPS 2 vs. 0

As shown in Table 2; Fig. 2, a total of 20 studies evaluated the association between the level of the mGPS and OS for pancreatic cancer patients. The mGPS ranged from 0 to 2 based on the CRP and albumin levels as discussed above. Since 12 cohorts divided the participants into 2 groups (high vs. low, mGPS = 1 as the cutoff value), and other participants in another 8 cohorts were grouped into 3 groups (mGPS = 0, 1 and 2), we separately performed a meta-analysis for different groupings. When divided into two groups, we defined an mGPS of 0 as the low group and an mGPS of 1 or 2 as the high group. There was evidence for moderate heterogeneity among studies (I^2^ = 61.7% and P = 0.003), so random-model was applied. The results indicated the statistically significant relationship between the mGPS and prognosis of patients with pancreatic cancer, and the OS may be better for patients with lower mGPS compared with patients with higher mGPS (HR = 1.50, 95% CI 1.20–1.89, P < 0.0001) (Fig. 2a). When divided into three groups, the random-effect model was applied due to significant heterogeneity (I^2^ = 86.6% and I^2^ = 80.4% for mGPS = 1 vs. mGPS = 0 and mGPS = 2 vs. mGPS = 0, respectively). The results also demonstrated a statistically significant difference between the high mGPS and poor survival for pancreatic cancer patients (mGPS = 1 vs. mGPS = 0: HR = 1.68, 95% CI 1.25–2.27, P = 0.001; mGPS = 2 vs. mGPS = 0: HR = 1.90, 95% CI 1.36–2.67, P < 0.0001, Fig. 2b–c).

**Table 2:** Overall and subgroup meta-analyses of the relationship between mGPS and OS

Subgroups	Studies/N	Patients/N	Pooled HR (95% CI, P)	Heterogeneity (P, I^²^, Model)
mGPS: high vs. low				
Overall	12	2009	1.504, (1.197–1.891), < 0.0001	0.003, 61.7%, random
Region				
East	9	1668	1.597, (1.240–2.056), < 0.0001	0.071, 44.7%, fixed
West	3	341	1.342, (0.797–2.258), 0.268	0.002, 83.8%, random
Therapy				
Chemotherapy	7	932	1.455, (1.048–2.020), 0.025	0.001, 74.2%, random
Pancreatectomy	5	1077	1.638, (1.247–2.150), < 0.0001	0.345, 10.7%, fixed
mGPS: 1 vs. 0				
Overall	8	4503	1.683, (1.247–2.269), 0.001	< 0.0001, 86.6%, random
Region				
East	7	4401	1.711, (1.248–2.347), 0.001	< 0.0001, 88.5%, random
West	1	102	1.346, (0.585–3.098), 0.485	–
Therapy				
Chemotherapy	6	2968	1.780, (1.297–2.443), < 0.0001	< 0.0001, 83.9%, random
Pancreatectomy	2	1535	1.521, (0.602–3.838), 0.375	0.001, 91.4%, random
mGPS: 2 vs. 0				
Overall	8	4503	1.899, (1.356–2.660), < 0.0001	< 0.0001, 80.4%, random
Region				
East	7	4401	1.833, (1.285–2.615), 0.001	< 0.0001, 82.3%, random
West	1	102	2.712, (1.252–5.875), 0.011	–
Therapy				
Chemotherapy	6	2968	1.872, (1.174–2.984), 0.008	< 0.0001, 81.5%, random
Pancreatectomy	2	1535	2.011, (0.941–4.297), 0.078	0.003, 88.3%, rrandom

“–” means not available; N means number

### Subgroup analyses of the association between mGPS and OS

In view of moderate heterogeneity among studies, we conducted subgroup analyses for OS by factors of the therapeutic method and study region, and the heterogeneity partly decreased in several subgroups. Detailed results of subgroup analyses are summarized in Table 2; Fig. 2. In 12 studies that chose mGPS = 1 as the cutoff value, the subgroup analyses based on the region showed that patients in eastern areas with higher mGPS had a decline of OS (HR = 1.60, 95% CI 1.24–2.06, P < 0.0001), which was not observed in patients in western areas (HR = 1.34, 95% CI 0.80–2.26, P = 0.268; Fig. 2d). Patients with higher mGPS in another 4 studies also demonstrated poor OS in eastern areas (mGPS = 1 vs. mGPS = 0, HR = 1.71, 95% CI 1.25–2.35, P = 0.001 and mGPS = 2 vs. mGPS = 0, HR = 1.83; 95% CI 1.29–2.62, P = 0.001; Fig. 2e–f). Further subgroup analyses based on treatment regimen showed that higher level of mGPS was significantly associated with worse OS in patients receiving chemotherapy (HR = 1.45, 95% CI 1. 05–2.02, P = 0.025) as well as pancreatectomy (HR = 1.64, 95% CI 1.25–2.15, P < 0.0001) in 12 studies that chose mGPS = 1 as the cutoff value (Fig. 2g). However, as for the 8 studies in which patients were divided into 2 groups (mGPS = 1 vs. mGPS = 0 and mGPS = 2 vs. mGPS = 0; Fig. 2h–i), the stratified analysis by the factor of therapeutic methods indicated that higher mGPS was linked to the poor OS in patients receiving chemotherapy (HR = 1.78, 95% CI 1.30–2.44, P < 0.0001 and HR = 1.87, 95% CI 1.17–2.98, P = 0.008, respectively), but not in patients undergoing pancreatectomy (HR = 1.52, 95% CI 0.60–3.84, P = 0.375 and HR = 2.01, 95% CI 0.94–4.30, P = 0.078, respectively).

### Publication bias and Influence analyses

**Fig. 3:**
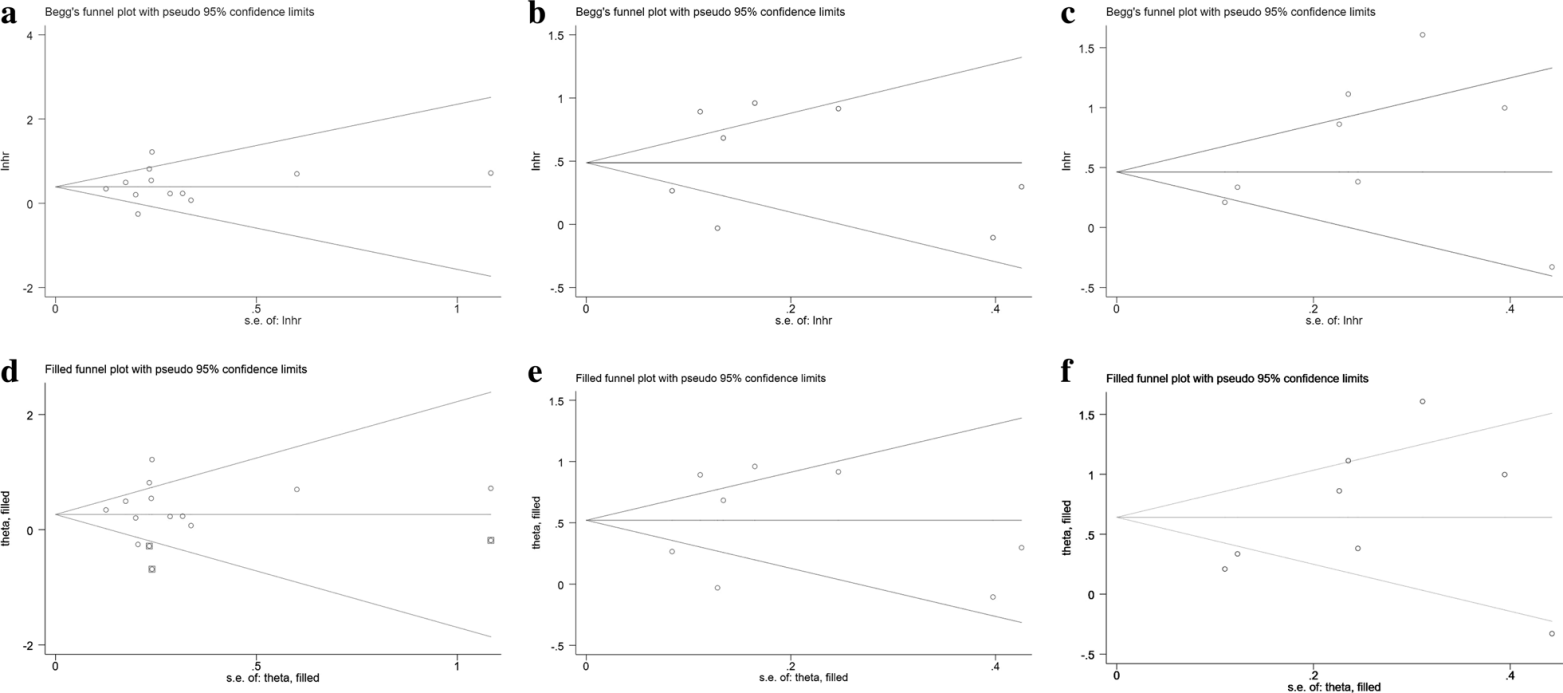
Publication bias of studies enrolled in the present meta-analysis. The Begg’s funnel plot: **a** mGPS high vs. low; **b** mGPS 1 vs. 0; **c** mGPS 2 vs. 0; Trim and fill analysis: **d** mGPS high vs. low; **e **mGPS 1 vs. 0; **f** mGPS 2 vs. 0

**Fig. 4:**
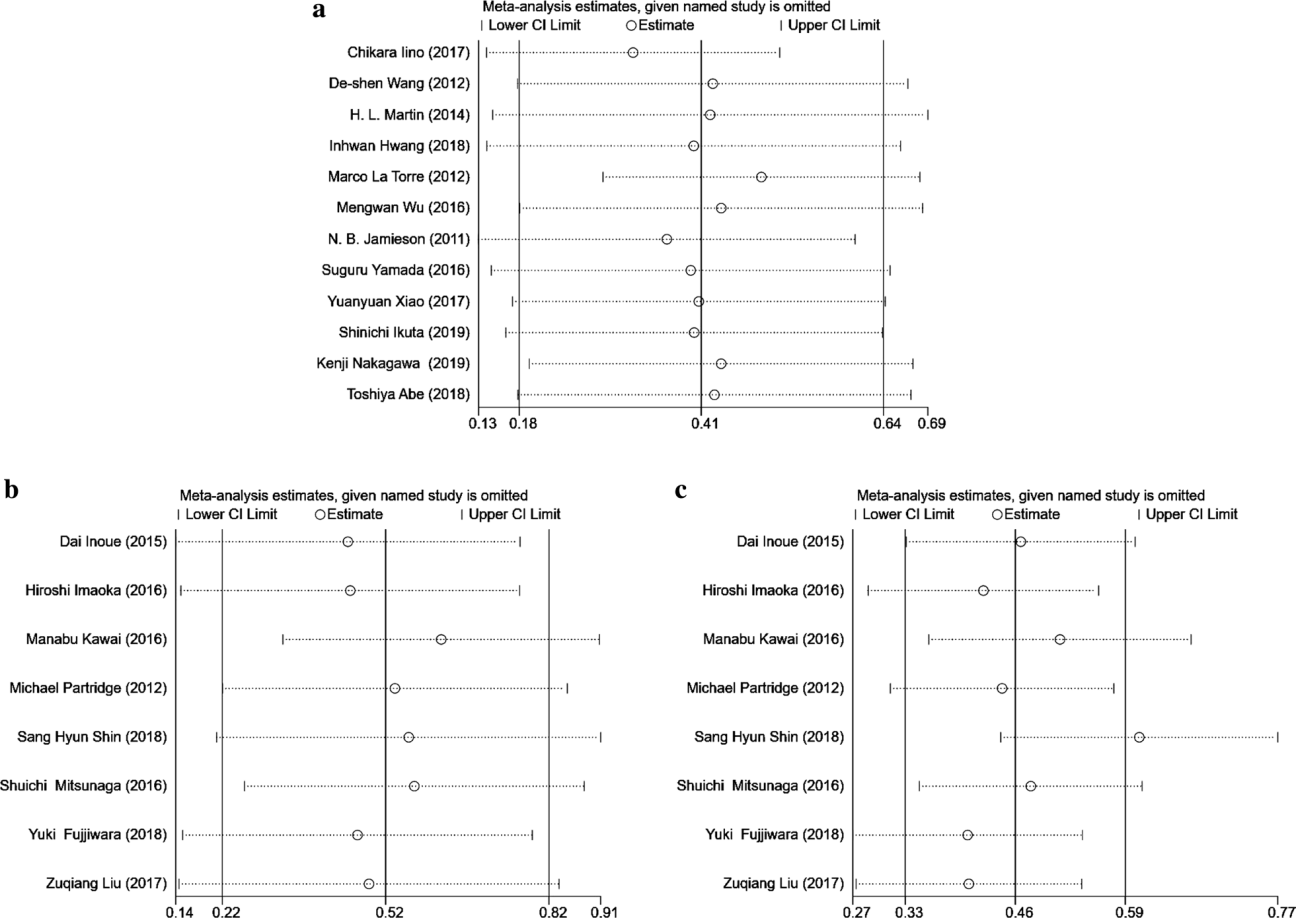
Influence analysis of studies enrolled in the present meta-analysis. **a** mGPS high vs. low; **b** mGPS 1 vs. 0; **c** mGPS 2 vs. 0

The Begg’s funnel plots seemed to be symmetrical, suggesting the absence of significant publication bias in all overall meta-analyses (Fig. 3a–c). The Begg’s test linear regression test also proved that there was no significant publication bias (each P > 0.05). Using trim and fill analysis, we only found that 3 studies evaluating the prognostic role of mGPS in OS in pancreatic cancer remained unpublished when participants were divided into 2 groups (high vs. low, mGPS = 1 as the cutoff value, Fig. 3d–f). The filled meta-analytic results for OS (pooled HR = 1.31, 95% CI [1.01–1.69], P < 0.001) also supported our original results. To examine the stability of the pooled HRs in OS, influence analysis was carried out with the successive omission of each study. The leaving-one-out study revealed that there was no individual cohort influencing the results greatly (Fig. 4a–c).

## Discussion

Pancreatic cancer is one of the most lethal cancer, and even pancreatic cancer in resectable stage shows a five-year survival rate of only 15–25% [39]. Unfortunately, 80–85% of patients present with advanced unresectable disease and pancreatic cancer responds poorly to most chemotherapeutic agents [40]. Therefore, it is essential to find a simple but effective way to help clinicians assess the prognosis of pancreatic cancer patients and choose the most appropriate treatment. Recently, mGPS has been suggested as a promising prognostic indicator in various cancers including pancreatic cancer.

This meta-analysis was the first to summary all eligible studies including 6512 patients to assess the prognostic value of the mGPS in patients with pancreatic cancer. After we controlled for other individual and clinical variables, the results showed that higher mGPS was closely linked with inferior OS. In view of certain heterogeneity among studies, we next conducted subgroup analysis for OS by factors of the therapeutic method and study regions. In addition, our findings suggested the prognostic value of mGPS as an independent prognostic factor for pancreatic cancer. In spite of remaining heterogeneity after subgroup analysis, it was partly reduced in some subgroups. We also carried out influence analysis to explore the source of heterogeneity, and there was no significant change in the trend of the adjusted results. Additionally, the absence of detective publication bias in our mate-analysis indicated that our research was credible, and the trim and fill analysis also supported original results.

Currently, mounting evidence reveals local immune response and systemic inflammation play a critical role in tumor growth, metastasis, and survival of cancer patients [5, 41]. As one of ten hallmarks of cancer, inflammatory cytokines produce by both the tumor and associate host cells affect tumor characteristics, including proliferation and survival of malignant cells, angiogenesis, metastasis, subversion of adaptive immunity, reduce response to hormones and chemotherapeutic agents [42, 43]. Consequently, systemic inflammatory indicators are extensively used to predict the recurrence and survival in pancreatic cancer patients after treatment [44]. Serum CRP is a typical acute-phase reactant mostly synthesized by the liver, induced by proinflammatory cytokines especially interleukin-6 (IL-6) [45]. Moses’s study has suggested that stimulated peripheral blood mononuclear cells from advanced pancreatic cancer patients usually produce high levels of IL-6 [46]. Accordingly, there may be a correlation between tumor-related inflammation and invasive tumor behavior, which leads to poor prognosis. The mechanism of acute-phase protein response in cancer patients is not clear. A plausible explanation is that the worsening disease may lead to more severe tumor-associated inflammation and tumor necrosis. Consequently, serum levels of CRP may merely reflect the tumor load of cancer patients. In addition, an acute-phase response may reflect a host-specific immune response to the tumor, or it may be as a consequence of the direct production of cytokines by tumor cells [47]. Serum level of albumin is also one of the most popular indicators of nutritional status, generally applied to evaluate the nutritional status, severity of disease and disease progression and prognosis [44]. Hypoalbuminemia usually occurs in combination with poor performance status, weight loss and nutritional deficiency, which negatively affect the prognosis of cancer patients [48]. In inflammatory states, hypoalbuminemia may result from reduced albumin synthesis or degradation [47, 49]. Accordingly, increased levels of inflammatory cytokines in tumors increase the demand for amino acids, resulting in the decreased serum albumin levels of patients with cachexia. Moreover, these cytokines, including tumor necrosis factor (TNF), increase the transcapillary passage of albumin as well as the permeability of the microvasculature. As a consequence, serum levels of albumin will drop [50]. Fleck’s group has demonstrated an elevated albumin transcapillary escape rate in patients with either sepsis or cancer [51]. Consequently, there is simply slight or even no hypoalbuminemia in early stages of cancer, but as the disease progresses the albumin levels drop significantly and may serve as ideal indicators of prognosis of cancer [48].

Although the clinical significance of pre-operative nutritional and immunological factors in pancreatic cancer has remained controversial, it is reported that CRP and albumin levels are good prognostic indicators of pancreatic cancer on account of correlation with host inflammatory-nutritional status [52]. Actually, the mGPS calculated by serum level of CRP and albumin could provide more accurate and comprehensive prognostic information than CRP alone [50, 53]. Furthermore, a prognostic tool such as the mGPS is more reliable and reproducible. If several clinicians were requested to evaluate performance status on a patient, there would be a degree of variability. When presented with CRP and albumin and asked to calculate an mGPS, there would be a consensus. To sum up, the mGPS is such an influential prognostic indicator for OS in pancreatic cancer patients that it deserves calculating as a part of the routine in the cancer patient’s management. It enables clinicians and patients to make a more informed choice about the appropriateness of chemotherapy or radiotherapy in advanced cancer [10]. As a promising and reliable inflammatory indicator, the mGPS is expected to predict the prognosis of pancreatic cancer patients and contribute to clinical decision making. However, further validation based on large cohort studies are still necessary.

Nevertheless, it is necessary to note that our research work still has some limitations. Firstly, most included studies have been conducted in China or Japan, which limits generalizability to some extent. Secondly, we only searched studies published in English and studies in other languages were neglected, which might cause selection bias and influenced the pooled results. Thirdly, most eligible studies are retrospective studies. Thus, potential publication bias may exist due to unpublished data with negative results, which might lead to overestimations in the pooled results. Lastly, there still exists moderate heterogeneity in this meta-analysis after the subgroup analysis, which may result from some confounding factors such as different disease progression of patients, tumor stage and sample size. Furthermore, we find no significant correlation between mGPS and OS in some subgroup analyses, which may demonstrate the potential influence of the region and treatment method in the prognostic value of mGPS in pancreatic cancer.

## Conclusion and future perspective

In summary, this meta-analysis is the first to demonstrate the close association between high level of mGPS and poor prognosis in pancreatic neoplasms. Besides, our meta-analysis suggests that the mGPS might serve as a novel and promising inflammatory prognostic indicator. More importantly, the mGPS derived from routine blood test could be used as a risk factor to stratify advanced pancreatic cancer patients into groups with different survival probabilities, which will better guide and optimize clinical decision-makings.

## Data Availability

The authors declare that all data supporting the findings of this study are available within the article and the enrolled articles for meta-analysis.

## Abbreviations

mGPS: Modified Glasgow Prognostic Score
NLR: Neutrophil-to-lymphocyte ratio
PLR: Platelet-to-lymphocyte ratio
CAR: C-reactive protein-to-albumin ratio
CRP: C-reactive protein
PRISMA: Preferred reporting items for systematic review and meta-analysis
OS: Overall survival
HR: Hazard ratio
CI: Confidence interval
NOS: Newcastle–Ottawa Scale
SE: Standard error
TNFF: Tumor necrosis factor
